# Bridging the Intention–Behavior Gap in Hotel Guests’ Pro-Environmental Behavior

**DOI:** 10.3390/bs16091549

**Published:** 2026-09-01

**Authors:** Thanh Mai Nguyen, Yunho Ji

**Affiliations:** Department of Tourism Administration, Kangwon National University, Chuncheon 24341, Republic of Korea; mai.nguyen.10998@gmail.com

**Keywords:** pro-environmental behavior, hotel industry, implementation intention, environmental knowledge, situational context

## Abstract

This study examines the relationships among hotel guests’ pro-environmental attitudes, behavioral intentions, implementation intentions, and pro-environmental behavior, while also investigating the moderating roles of environmental knowledge and situational context. Data were collected through a self-administered survey of 204 domestic Korean tourists who had stayed at hotels or resorts in Gangwon State, South Korea, and analyzed using SPSS 27, PROCESS Macro Model 4, and hierarchical regression analysis. The results showed that implementation intention was positively associated with pro-environmental behavior and partially mediated the relationships of attitude and behavioral intention with behavior. Environmental knowledge strengthened the relationship between attitude and implementation intention but did not moderate the relationship between behavioral intention and implementation intention. Situational context had no significant moderating effect. These findings identify implementation intention as an important indirect mechanism associated with hotel guests’ pro-environmental behavior. Practically, hotels should provide situation-specific action prompts, clear recycling instructions, towel and linen reuse options, and accessible energy- and water-saving facilities to facilitate guests’ pro-environmental practices.

## 1. Introduction

Pro-environmental behavior refers to actions taken by individuals to reduce environmental harm and support conservation and sustainable development (Kollmuss & Agyeman, 2002). As environmental concerns have increased, tourists have shown greater interest in environmental protection and responsible travel behavior (Bremner, 2020). For example, Booking.com (2024) reported that 83% of global tourists consider sustainable travel important and 75% intend to travel more sustainably within the next 12 months. Similarly, according to Greeneration (2025), the World Tourism Organization (UNWTO)’s 2023 Sustainable Tourism Report indicated that 73% of global tourists prefer eco-friendly hotels, a proportion that has continued to increase in recent years (Greeneration, 2025).

Despite this growing environmental awareness and positive behavioral intention, consumers’ intentions do not always translate into actual behavior. This inconsistency is commonly referred to as the intention–behavior gap in pro-environmental behavior (Carrero et al., 2025). In consumer behavior research, most studies on pro-environmental behavior have primarily examined the relationship between attitudes toward such behavior and behavioral intentions (Wang & Mangmeechai, 2021; Tawde et al., 2023). However, this line of research has been criticized for oversimplifying the complex process through which intentions are converted into actual behavior (Carrington et al., 2010; Grimmer & Miles, 2017). The theory of planned behavior, a widely used framework in studies of consumers’ pro-environmental behavior, has been criticized for its limited explanatory power because attitudes and intentions may be unstable and the theory does not sufficiently account for specific action plans required for behavioral implementation (Hassan et al., 2016). In addition, a meta-analysis of 185 independent studies found that the theory of planned behavior explained only 27% of the variance in behavior (Armitage & Conner, 2001). Therefore, to better understand why consumers do not always engage in pro-environmental behavior, it is necessary to focus more closely on the intention–behavior gap (Carrington et al., 2010).

Pro-environmental behavior is shaped not only by individuals’ attitudes and intentions, but also by cognitive factors and external contextual conditions. Therefore, a more comprehensive understanding of how pro-environmental behavior is formed requires consideration of these factors. Environmental knowledge refers to individuals’ understanding of environmental problems and their awareness of ways to reduce negative environmental impacts (Cuong, 2025; Muna et al., 2025). It can be viewed as an important factor that facilitates the practice of pro-environmental behavior (Pothitou et al., 2016; Kollmuss & Agyeman, 2002). In addition, pro-environmental behavior is not determined solely by consumers’ attitudes or intentions, but may also be influenced by situational context of which consumers are not consciously aware (Carrington et al., 2010). Situational context is thus an important element that affects consumers’ decision-making and behavior formation processes, suggesting the need to consider the specific context in which consumption behavior occurs (Belk, 1975; Carrington et al., 2010; Richter & Klöckner, 2017; Wu et al., 2021).

Hotels represent a unique consumption context in which pro-environmental behavior may differ from that in everyday life. Although guests may hold favorable pro-environmental attitudes and intentions, their behavior can be influenced by contextual factors such as convenience, time constraints, social interactions, and the hotel environment (Mbasera et al., 2016; Abdou et al., 2022). Previous studies have shown that consumers tend to engage in pro-environmental behavior less frequently in hotels than in their daily lives (Miao & Wei, 2013). This suggests that the formation and enactment of pro-environmental behavior in hotel settings may follow a process different from that observed in everyday contexts. However, although previous hotel studies have extensively examined the antecedents of pro-environmental intentions, limited attention has been paid to the post-intentional process through which attitudes and behavioral intentions develop into situation-specific action plans and are subsequently associated with behavior. Moreover, few hotel studies have examined implementation intention together with environmental knowledge and situational context within an integrated framework. Therefore, understanding hotel guests’ pro-environmental behavior requires considering not only attitudes and behavioral intentions but also the post-intentional role of implementation intention and the relationship-specific influences of environmental knowledge and situational context.

Against this background, this study examines the formation of hotel guests’ pro-environmental behavior, with particular attention to the mediating role of implementation intention and the moderating roles of environmental knowledge and situational context. Accordingly, the study addresses the following research questions: (1) How are attitude, behavioral intention, implementation intention, and pro-environmental behavior related among hotel guests? (2) Does implementation intention mediate the relationships between attitude and pro-environmental behavior and between behavioral intention and pro-environmental behavior? (3) Does environmental knowledge moderate the relationships of attitude and behavioral intention with implementation intention, and does situational context moderate the relationship between implementation intention and pro-environmental behavior? This study makes two main contributions. First, it extends hotel pro-environmental behavior research beyond the conventional attitude–intention relationship by examining the post-intentional process through which attitudes and behavioral intentions are indirectly associated with pro-environmental behavior via implementation intention. Second, by examining the relationship-specific moderating roles of environmental knowledge and situational context, the study provides a more comprehensive account of how individual cognitive factors and external conditions are associated with hotel guests’ pro-environmental behavior. In doing so, the study contributes to research on the intention–behavior gap and offers practical insights for hotel managers seeking to facilitate environmentally responsible guest behavior.

## 2. Review of the Literature

### 2.1. Pro-Environmental Behavior

Pro-environmental behavior refers to voluntary actions undertaken by individuals to minimize negative impacts on the environment and contribute to environmental conservation and sustainable development (Kollmuss & Agyeman, 2002). Such behavior is increasingly recognized as playing an important role not only in addressing environmental problems, but also in promoting economic and social sustainability and improving the quality of life of future generations (Blankenberg & Alhusen, 2019; Karimi et al., 2022). In the tourism and hospitality context, pro-environmental behavior encompasses a wide range of actions through which tourists and hotel guests seek to reduce their environmental impact, including choosing eco-friendly hotels, conserving energy, reusing towels and bed linens, and reducing water consumption (Sousa et al., 2025). Pro-environmental behavior among hotel guests is particularly important because it not only helps reduce environmental impact but also supports hotels’ efforts to implement sustainable management practices (Damijanić et al., 2023).

Meanwhile, pro-environmental behavioral intention has been regarded as a key antecedent of actual pro-environmental behavior, while attitude toward a behavior has been identified as an important determinant of behavioral intention (Ajzen, 1991). According to the theory of planned behavior, individuals who hold more favorable attitudes toward a particular behavior are more likely to develop a stronger willingness to perform that behavior, which ultimately leads to the formation of behavioral intention (Ajzen, 1991). A positive relationship between attitude and behavioral intention has also been consistently documented in studies of pro-environmental behavior. In the context of everyday pro-environmental behavior, attitude has been found to be an important factor that strengthens pro-environmental behavioral intention (Ates, 2020; P. Liu et al., 2020; Sultana et al., 2020; Karimi et al., 2022; Batool et al., 2024). Similar findings have also been reported in the hotel and tourism literature. Han et al. (2010) and Han (2015) showed that favorable attitudes toward green hotels increase consumers’ intention to choose green hotels, while Trang et al. (2019) confirmed that attitude strengthens behavioral intention in relation to visiting green hotels and engaging in pro-environmental behavior during hotel stays. In addition, Budovska et al. (2020) reported that hotel guests’ attitudes positively influence their intention to reuse towels, and Liang et al. (2025) also found that hotel guests’ pro-environmental attitudes have a positive effect on their intention to engage in pro-environmental behavior in hotels. Although prior studies consistently support the positive relationship between attitude and behavioral intention, most hotel research has focused on intention formation rather than the post-intentional process leading to behavior. Based on the above discussion, the following hypothesis is proposed.

**Hypothesis** **1.** *Attitude toward pro-environmental behavior is positively associated with pro-environmental behavioral intention.*

### 2.2. The Formation of Pro-Environmental Behavior and the Role of Implementation Intention

Implementation intention is a self-regulatory construct that transforms a general behavioral intention into a specific action plan for a particular situation. Whereas behavioral intention refers to an individual’s overall willingness or motivational readiness to perform a behavior, implementation intention specifies when, where, and how that behavior will be carried out (Grimmer & Miles, 2017). It is commonly expressed in an if–then format, such as “If situation Y occurs, then I will perform behavior Z” (Wieber et al., 2015; Wang & Mangmeechai, 2021), although it may also take the form of a more detailed action plan depending on the nature and complexity of the behavior (Adam & Fayolle, 2016; Carrero et al., 2025). Implementation intention is conceptually distinct from behavior because it represents a future-oriented, situation-specific action plan, whereas behavior refers to the enactment of an action. In this sense, behavioral intention reflects the willingness to act, implementation intention reflects a situation-specific plan for action, and behavior reflects the enactment of that action. By linking a situational cue with a corresponding behavioral response in advance, implementation intention converts a general intention into a more concrete action plan and increases the likelihood that the planned response will be initiated when the relevant cue is encountered (Gollwitzer & Sheeran, 2006; Webb & Sheeran, 2007).

Even when individuals form behavioral intentions, these intentions often fail to translate into actual behavior (Sheeran, 2002). Implementation intention has therefore been introduced as an important construct for explaining the intention–behavior gap, drawing on the Rubicon Model of Action Phases (Heckhausen & Gollwitzer, 1987; Achtziger & Gollwitzer, 2018) and the theory of implementation intention (Gollwitzer, 1993, 1999). Gollwitzer (1993, 1999) described implementation intention as a self-regulatory strategy that helps individuals overcome the difficulties that may arise when turning behavioral intentions into action. According to the Rubicon Model, individuals first evaluate the desirability and feasibility of a behavior, set a goal, and then develop a specific plan for carrying out the intended behavior (Achtziger & Gollwitzer, 2018; Asenkerschbaumer et al., 2024). In this regard, implementation intention serves as a key mechanism that links general behavioral intention to actual behavior.

A growing body of research has identified implementation intention as a key mediating mechanism that explains how behavioral intention is translated into behavior (Rise et al., 2003; Dholakia et al., 2007; Scholz et al., 2008; Carrington et al., 2010; Hassan et al., 2016). Carrington et al. (2010) proposed the Model of the Intention–Behaviour Gap of Ethically Minded Consumers, in which implementation intention mediates the relationship between behavioral intention and actual behavior. This perspective suggests that favorable intentions alone may not be sufficient to produce behavior unless they are converted into concrete plans for action. Building on this framework, Grimmer and Miles (2017) demonstrated that pro-environmental purchase intention influences actual pro-environmental behavior indirectly through implementation intention. Similar mediating effects were reported by Wang and Mangmeechai (2021) and Tawde et al. (2023), indicating that implementation intention serves as an important mechanism linking general behavioral motivation to subsequent pro-environmental action. In addition, Wang et al. (2021) found that attitudes toward pro-environmental behavior positively influence implementation intention, which in turn contributes to actual behavior. Taken together, these findings indicate that implementation intention should not be viewed merely as an additional predictor of behavior. Although previous studies have examined it as both a direct predictor and a mediator, these roles are complementary: implementation intention may be directly associated with behavior while also transmitting the associations of upstream motivational factors with behavior. By positioning implementation intention after attitude and behavioral intention, the proposed framework extends the explanatory scope of selected TPB relationships beyond motivational intention formation to situation-specific action planning. This integration contributes theoretically by identifying a post-intentional mechanism through which attitudes and behavioral intentions are indirectly associated with pro-environmental behavior. However, these mediating relationships remain insufficiently examined in the context of hotel guests. Based on the previous literature, the following hypotheses are proposed:

**Hypothesis** **2.** *Attitude toward pro-environmental behavior is positively associated with implementation intention for pro-environmental behavior.*

**Hypothesis** **3.** *Pro-environmental behavioral intention is positively associated with implementation intention for pro-environmental behavior.*

**Hypothesis** **4.** *Implementation intention is positively associated with pro-environmental behavior.*

**Hypothesis** **5.** *Implementation intention mediates the association between attitude toward pro-environmental behavior and pro-environmental behavior.*

**Hypothesis** **6.** *Implementation intention mediates the association between pro-environmental behavioral intention and pro-environmental behavior.*

### 2.3. Environmental Knowledge

Environmental knowledge refers to the extent to which individuals understand facts, concepts, impacts, and possible solutions related to environmental issues (Damijanić et al., 2023; Muna et al., 2025). It includes knowledge that enables individuals to evaluate the environmental consequences of specific behaviors or products (Chaihanchanchai & Anantachart, 2023), and is regarded as an important cognitive factor that fosters a sense of environmental responsibility and encourages responsible behavior (Cuong, 2025). By providing an understanding of environmental problems and enabling individuals to use relevant information, environmental knowledge encourages participation in environmental protection activities and has been widely recognized as a key determinant of pro-environmental behavior (Dursun et al., 2019; Liobikiene & Poškus, 2019; P. Liu et al., 2020). Conversely, individuals with insufficient environmental knowledge may have difficulty engaging in pro-environmental behavior because they lack an adequate understanding of environmental problems and possible solutions (Muna et al., 2025; Cuong, 2025). In the tourism and hospitality context, tourists with higher levels of environmental knowledge have also been found to be more likely to participate in pro-environmental activities (M. Kim et al., 2018; Cuong, 2025).

Previous studies have identified environmental knowledge as an important cognitive factor that helps individuals translate pro-environmental attitudes and intentions into more concrete forms of behavioral preparation. In their meta-analysis, Hines et al. (1987) emphasized that knowledge of environmental problems and possible solutions provides individuals with the information needed to identify appropriate environmental actions. Subsequent empirical studies have similarly shown that environmental knowledge is closely associated with environmental attitudes, behavioral intentions, and pro-environmental behavior by enhancing individuals’ understanding of the consequences and appropriate courses of environmental action (Liobikiene & Poškus, 2019; P. Liu et al., 2020; Pothitou et al., 2016). In the tourism and hospitality context, such knowledge may be particularly relevant because tourists must translate their general environmental attitudes and intentions into specific behavioral choices within unfamiliar consumption settings; accordingly, environmental knowledge has been associated with stronger environmental attitudes and behavioral intentions (Cuong, 2025), pro-environmental purchasing behavior (M. Kim et al., 2018; Muna et al., 2025), and intentions to visit green hotels (Damijanić et al., 2023).

Meanwhile, environmental knowledge has also been found to serve as a moderator that strengthens the relationships among variables related to pro-environmental behavior (Joseph, 2019; Joshi & Rahman, 2015; Kollmuss & Agyeman, 2002). Wang et al. (2021) explained that individuals with higher levels of environmental knowledge are more likely to translate their implementation intentions into actual pro-environmental behavior. Similarly, Chaihanchanchai and Anantachart (2023) reported that environmental knowledge strengthens the relationship between green purchasing attitudes and purchasing behavior. These findings suggest that environmental knowledge is an important moderator that reinforces the relationships among key variables in the formation process of pro-environmental behavior. Environmental knowledge has been examined as both an antecedent and a moderator, but its moderating influence has been tested across different relationships and contexts. Therefore it remains unclear whether it strengthens the formation of implementation intention from attitude, behavioral intention, or both. Based on the previous literature, the following hypothesis is proposed:

**Hypothesis** **7-1.** *Environmental knowledge moderates the association between attitude toward pro-environmental behavior and implementation intention for pro-environmental behavior.*

**Hypothesis** **7-2.** *Environmental knowledge moderates the association between pro-environmental behavioral intention and implementation intention for pro-environmental behavior.*

### 2.4. Situational Context

Situational context refers to the various external conditions present at a specific time and place in which consumption behavior occurs. It is distinct from consumers’ personal characteristics, such as personality and attitudes, as well as from the attributes of the product itself (Belk, 1975; Karnowski & Jandura, 2014). In pro-environmental behavior research, situational context represents temporary environmental conditions that arise during the behavioral process and may either facilitate or hinder the translation of consumers’ behavioral intentions into actual behavior (Carrington et al., 2010). Consumer behavior is shaped not only by psychological factors but also by external conditions, as consumers interact with physical and social environments when translating intentions into actual behavior. Thus, the intention–behavior gap cannot be fully explained solely by individuals’ values or attitudes (Carrington et al., 2010). Moreover, for a behavior to be actually performed, individuals require not only personal motivation but also supportive environmental conditions (Richter & Klöckner, 2017). Previous studies have reported that various contextual factors, such as price, product availability, the socio-cultural environment, media communication, and legal and institutional systems, influence pro-environmental behavior (Kollmuss & Agyeman, 2002; Joshi & Rahman, 2015).

Previous studies have also confirmed that situational context plays an important role in determining whether pro-environmental intentions and plans are enacted as behavior. Grimmer et al. (2016) explained that contextual factors such as time, price, product availability, and ease of purchase can strengthen or weaken the relationships between purchase intention and implementation intention, as well as between implementation intention and actual behavior. Liao and Yang (2021) further found that situational constraints affect the extent to which pro-environmental intentions are translated into behavior, and that these effects may vary depending on the type of behavior. N. Kim and Lee (2023) likewise emphasized that contextual conditions such as ease of purchase and trust in eco-labels shape whether consumers are able to carry out environmentally responsible choices. These findings suggest that situational context functions as an external enabling or constraining condition that can influence the extent to which implementation intentions are translated into pro-environmental behavior. Although situational conditions may facilitate or constrain behavior, implementation intentions may reduce dependence on external conditions by linking situational cues to planned responses. Whether situational context still moderates the implementation intention–behavior relationship in hotel settings therefore remains unresolved. Based on the previous literature, the following hypothesis is proposed:

**Hypothesis** **8.** *Situational context moderates the association between implementation intention and pro-environmental behavior.*

## 3. Research Method

### 3.1. Conceptual Framework

This study examines the relationships underlying hotel guests’ pro-environmental behavior by drawing on selected constructs from the Theory of Planned Behavior and implementation intention theory, within the broader perspective of the intention–behavior gap. TPB provides the motivational starting point through attitude and behavioral intention, whereas implementation intention theory explains how general intentions are developed into situation-specific action plans. The intention–behavior gap represents the research problem motivating the examination of the post-intentional process. Accordingly, this study examines whether attitude and behavioral intention are indirectly associated with pro-environmental behavior through implementation intention. Given the cross-sectional design, the proposed relationships are interpreted as statistical associations rather than as a temporally ordered behavioral process. The study does not aim to test or extend the full TPB but selectively draws on attitude and behavioral intention as motivational antecedents. Subjective norms and perceived behavioral control were not included because the primary purpose was to examine implementation intention as a post-intentional self-regulatory mechanism. Thus, the proposed framework is positioned as a post-intentional implementation framework informed by selected TPB constructs.

In addition, pro-environmental behavior is known to be influenced not only by internal factors, such as individuals’ attitudes and intentions, but also by cognitive factors and external environmental conditions (Kollmuss & Agyeman, 2002; Carrington et al., 2010). Accordingly, this study conceptualizes environmental knowledge as an individual cognitive factor and examines whether it moderates the relationships between attitude and implementation intention, and between behavioral intention and implementation intention. Furthermore, based on the view that consumer behavior can be shaped by the situational context in which the behavior takes place (Belk, 1975; Richter & Klöckner, 2017), this study also examines whether hotel-related situational context moderates the relationship between implementation intention and pro-environmental behavior.

**Figure 1:**
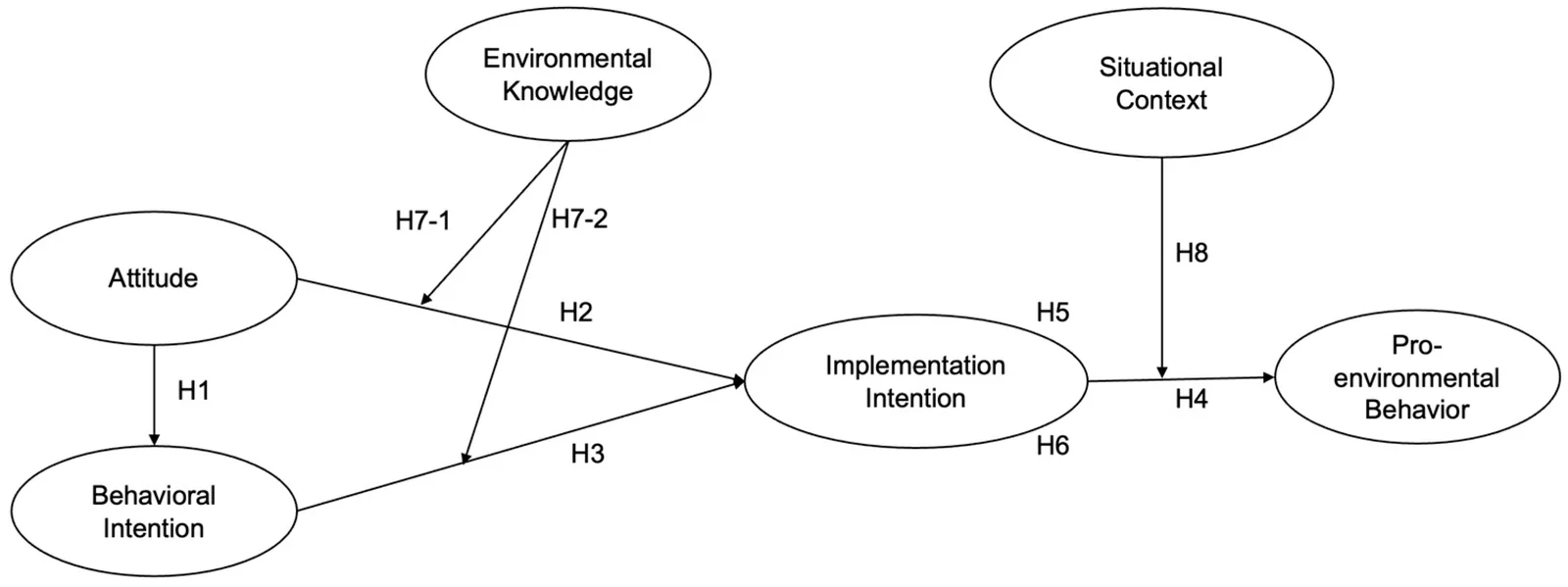
Research model.

The research model is presented in Figure 1.

### 3.2. Data Collection and Analysis

This study surveyed Korean domestic tourists who had stayed for at least one night at a four- or five-star hotel or resort in Gangwon State within the previous year. Gangwon State was selected because it is one of South Korea’s major tourism and leisure destinations, with a high concentration of mountain, coastal, and recreational hotels and resorts. These facilities are primarily used for leisure travel, making the region an appropriate setting for examining hotel guests’ pro-environmental behavior. Data were collected from 1 April to 17 April 2026. Respondents were first screened to confirm that they had stayed at a four- or five-star hotel or resort in Gangwon State within the previous year. Those who did not meet these criteria were excluded. The questionnaire was administered in Korean and self-administered in both the on-site and online surveys. The same quantitative questionnaire was administered through both online and on-site modes. Prior to completing the questionnaire, respondents were given a brief explanation of pro-environmental behavior in the hotel context. Data collection was conducted by the Leisure Tech Research Institute using a mixed-mode survey approach. Of the 230 questionnaires collected, 182 responses (79.1%) were obtained through on-site surveys and 48 responses (20.9%) were collected online. The same eligibility criteria were applied to both survey modes. After excluding 26 responses with missing values or insufficient response consistency, 204 valid responses were retained for the final analysis.

Data analysis was performed using SPSS 27. First, frequency analysis was conducted to examine the demographic characteristics of the respondents. To assess the validity and reliability of the measurement instruments, exploratory factor analysis and reliability analysis were carried out. Principal component analysis with Varimax rotation was applied for factor analysis, and the suitability of the data for factor analysis was evaluated using the Kaiser–Meyer–Olkin (KMO) measure and Bartlett’s test of sphericity (Hair et al., 2010). Factors were extracted based on the criterion of eigenvalues greater than 1.0, and factor loadings of 0.50 or higher were considered significant (Hair et al., 2010). In addition, Cronbach’s alpha coefficients were calculated to assess the internal consistency of the measurement variables, with values of 0.70 or higher regarded as acceptable (Hair et al., 2010). Because all major variables were measured from the same respondents using a self-reported questionnaire, Harman’s single-factor test was conducted to assess potential common method bias. All measurement items were entered into an unrotated exploratory factor analysis. Common method bias was considered potentially serious if a single factor emerged or if the first factor accounted for more than 50% of the total variance. Next, correlation analysis was conducted to examine the relationships among the variables, and simple and multiple regression analyses were performed to test the hypotheses. To formally examine the indirect effects among the variables, mediation analysis was additionally conducted using Hayes’ PROCESS Macro Model 4 for SPSS. Indirect effects were estimated using 5000 bootstrap samples and 95% bootstrap confidence intervals. An indirect effect was considered statistically significant when the confidence interval did not include zero. Finally, hierarchical regression analysis was used to examine the moderating effects of environmental knowledge and situational context. The moderating effects were assessed by sequentially entering the independent variable, the moderating variable, and the interaction term into the regression model, and by evaluating the increase in explanatory power (R^2^) and the significance of the change in F-values (Hair et al., 2010).

### 3.3. Operational Definitions and Measurement of Variables

The measurement items used in this study were adapted and refined from previous studies to fit the research context. Attitude toward pro-environmental behavior refers to individuals’ overall evaluation of such behavior, whereas behavioral intention refers to their general willingness to engage in pro-environmental behavior during future hotel stays. Implementation intention refers to a prospective if–then action plan specifying the situational conditions under which a particular behavior will be performed. Pro-environmental behavior refers to actions taken to minimize environmental impacts during hotel stays. In this study, pro-environmental behavior was operationalized as respondents’ self-reported behavior during their most recent hotel stay. Environmental knowledge refers to individuals’ understanding of environmental problems and possible solutions, while situational context refers to external conditions that may facilitate or constrain the relationship between implementation intention and pro-environmental behavior.

Each construct was measured with five items adapted from previous studies. Specifically, the items for attitude toward pro-environmental behavior were adapted from Han et al. (2010) and J. Liu et al. (2019), while behavioral intention was measured based on J. Liu et al. (2019) and Sousa et al. (2025). Implementation intention was measured using items adapted from Rise et al. (2003) and Sousa et al. (2025), and pro-environmental behavior was measured based on Miao and Wei (2013) and Sousa et al. (2025). The items for environmental knowledge were adapted from Damijanić et al. (2023) and Cuong (2025), whereas situational context was measured using items adapted from Carrington et al. (2010) and Grimmer and Miles (2017). All items were measured on a seven-point Likert scale ranging from 1 = strongly disagree to 7 = strongly agree.

The measurement items are presented in Table 1.

## 4. Results

### 4.1. Demographic Characteristics

A total of 204 valid responses were analyzed. In terms of gender, 115 respondents were male (56.4%) and 89 were female (43.6%). Regarding age, respondents in their 40s accounted for the largest proportion (39.2%), followed by those in their 50s (32.4%) and those aged 60 or above (15.2%). With respect to education level, the majority of respondents were university graduates (76.5%), followed by graduate school graduates (16.7%) and high school graduates (6.9%). Regarding average monthly household income, more than half of the respondents reported an income of 5 million KRW to less than 10 million KRW (52.0%), followed by 3 million KRW to less than 5 million KRW (23.0%). In terms of the number of hotel stays per year, the largest group had stayed at hotels three to five times (49.5%), followed by one to two times (27.0%) and six to nine times (19.6%). The main purpose of the most recent hotel stay was tourism and leisure (61.3%), followed by business and family gatherings, each accounting for 14.7%. Finally, regarding travel companions, the largest proportion of respondents stayed with friends or partners (54.4%), followed by family members (23.0%).

The demographic characteristics of the respondents are presented in Table 2.

### 4.2. Validity, Reliability, and Common Method Bias

Exploratory factor analysis and reliability analysis were conducted to assess the validity and reliability of the measurement instruments. The initial analysis showed that the factor loadings of pro-environmental behavioral intention item BI2, environmental knowledge item EK1, and situational context item SC5 were 0.430, 0.329, and 0.166, respectively, which were below the recommended threshold of 0.50. In addition, the overall Cronbach’s alpha coefficient for situational context was 0.607, indicating insufficient internal consistency. In particular, the reliability of the situational context construct was found to improve when item SC5 was removed. Therefore, BI2, EK1, and SC5 were excluded, and exploratory factor analysis and reliability analysis were conducted again.

The final analysis results are presented in Table 3. As shown in the table, the KMO values for all constructs were above 0.80, and Bartlett’s test of sphericity was statistically significant (*p* < 0.05). In addition, the eigenvalues of all factors exceeded 1.0, and all measurement items showed factor loadings of approximately 0.70 or higher, indicating that convergent validity was established (Hair et al., 2010). The reliability analysis also showed that Cronbach’s alpha coefficients for all constructs were above 0.80, confirming sufficient internal consistency and reliability of the measurement instruments. Therefore, the measurement scales used in this study were considered to have adequate validity and reliability. The final Cronbach’s alpha coefficients ranged from 0.865 to 0.936. Because several coefficients exceeded 0.90, additional item-level reliability checks were conducted. The corrected item–total correlations ranged from 0.700 to 0.841, and deleting any retained item reduced the Cronbach’s alpha coefficient of the corresponding scale.

Harman’s single-factor test was conducted to assess potential common method bias. The unrotated exploratory factor analysis extracted four factors, and the first factor explained 37.001% of the total variance. Because this value was below the commonly used threshold of 50%, common method bias was not considered a dominant concern. Nevertheless, because all variables were collected through a cross-sectional self-reported survey, the possibility of common method bias cannot be completely excluded.

### 4.3. Correlation and Discriminant Validity Analysis

Pearson correlation analysis was conducted to examine the relationships among the main variables, and the results are presented in Table 4. Attitude toward pro-environmental behavior was positively correlated with behavioral intention (r = 0.471, *p* < 0.01), implementation intention (r = 0.456, *p* < 0.01), and pro-environmental behavior (r = 0.345, *p* < 0.01). Behavioral intention was also positively correlated with implementation intention (r = 0.645, *p* < 0.01) and pro-environmental behavior (r = 0.465, *p* < 0.01). In addition, implementation intention was positively correlated with pro-environmental behavior (r = 0.449, *p* < 0.01). Environmental knowledge showed positive correlations with attitude (r = 0.251, *p* < 0.01), behavioral intention (r = 0.347, *p* < 0.01), implementation intention (r = 0.431, *p* < 0.01), and pro-environmental behavior (r = 0.308, *p* < 0.01). Situational context was positively correlated with behavioral intention (r = 0.300, *p* < 0.01), implementation intention (r = 0.326, *p* < 0.01), pro-environmental behavior (r = 0.256, *p* < 0.01), and environmental knowledge (r = 0.448, *p* < 0.01). However, the relationship between situational context and attitude was not statistically significant (r = 0.095, *p* > 0.05).

Discriminant validity was assessed using the Fornell–Larcker criterion. The AVE values ranged from 0.564 to 0.726, exceeding the recommended threshold of 0.50. In addition, the square root of the AVE for each construct was greater than its correlations with the other constructs. In particular, although behavioral intention and implementation intention showed the highest correlation (r = 0.645), this value was lower than the square roots of their respective AVE values, 0.765 and 0.751. The correlation between implementation intention and pro-environmental behavior (r = 0.449) was also lower than their corresponding square roots of the AVE, 0.751 and 0.835. These results indicate adequate discriminant validity among the constructs.

Furthermore, all correlation coefficients were below 0.70, suggesting that multicollinearity was unlikely to be a serious concern. Overall, the relationships among the variables were generally consistent with the proposed hypotheses.

### 4.4. Hypothesis Testing

#### 4.4.1. Effect of Attitude Toward Pro-Environmental Behavior on Behavioral Intention

To test Hypothesis 1, simple regression analysis was conducted with attitude toward pro-environmental behavior as the independent variable and pro-environmental behavioral intention as the dependent variable. The results are presented in Table 5. The explanatory power of the regression model was 22.2% (R^2^ = 0.222), and the adjusted coefficient of determination (Adj. R^2^) was 0.218. The F-value of the regression model was 57.658 (*p* < 0.001), indicating that the overall model was statistically significant.

The standardized regression coefficient (β) for attitude toward pro-environmental behavior was 0.471 (*p* < 0.001), confirming that attitude toward pro-environmental behavior had a significant positive effect on pro-environmental behavioral intention. This indicates that the more positively hotel guests evaluate pro-environmental behavior, the stronger their willingness becomes to engage in such behavior during hotel stays. Therefore, Hypothesis 1 was supported.

#### 4.4.2. Effects of Attitude Toward Pro-Environmental Behavior and Behavioral Intention on Implementation Intention

To test hypotheses 2 and 3, multiple regression analysis was conducted with attitude toward pro-environmental behavior and behavioral intention as the independent variables, and implementation intention as the dependent variable. The results are presented in Table 5. The explanatory power of the regression model was 44.6% (R^2^ = 0.446), and the adjusted coefficient of determination (Adj. R^2^) was 0.440. The F-value of the regression model was 80.861 (*p* < 0.001), indicating that the overall model was statistically significant.

First, the results for Hypothesis 2 showed that attitude toward pro-environmental behavior had a significant positive effect on implementation intention (β = 0.196, *p* = 0.001). This indicates that the more positive hotel guests’ attitudes are toward pro-environmental behavior, the more likely they are to develop specific action plans for engaging in such behavior. Therefore, Hypothesis 2 was supported.

Next, the results for Hypothesis 3 confirmed that behavioral intention had a significant positive effect on implementation intention (β = 0.553, *p* < 0.001). This suggests that the stronger hotel guests’ willingness is to engage in pro-environmental behavior, the more likely they are to formulate specific plans for translating this intention into actual behavior. Therefore, Hypothesis 3 was also supported.

Meanwhile, comparison of the standardized regression coefficients showed that behavioral intention (β = 0.553) had a stronger effect on implementation intention than attitude (β = 0.196). This suggests that although positive evaluations of pro-environmental behavior are important, the formation of a willingness to act is more likely to develop into implementation plans. In other words, implementation intention appears to be more closely associated with behavioral intention than with attitude, indicating that it may function as a key mediating process linking behavioral intention to actual behavior.

#### 4.4.3. Relationship Between Implementation Intention and Pro-Environmental Behavior

To test Hypothesis 4, simple regression analysis was conducted with implementation intention for pro-environmental behavior as the independent variable and pro-environmental behavior as the dependent variable. The results are presented in Table 5. The explanatory power of the regression model was 20.1% (R^2^ = 0.201), and the adjusted coefficient of determination (Adj. R^2^) was 0.198. The F-value of the regression model was 50.973 (*p* < 0.001), indicating that the overall model was statistically significant.

The analysis showed that the standardized regression coefficient for implementation intention for pro-environmental behavior was 0.449 (*p* < 0.001), confirming that implementation intention was positively associated with pro-environmental behavior. This indicates that the more specifically hotel guests plan to engage in pro-environmental behavior, the more likely they are to perform such behavior during their hotel stays. Therefore, Hypothesis 4 was supported.

#### 4.4.4. Mediating Effect of Implementation Intention

To test Hypotheses 5 and 6, mediation analysis was conducted using Hayes’ PROCESS Macro Model 4 for SPSS. Two separate models were examined to assess whether implementation intention mediated the relationships of attitude and behavioral intention with pro-environmental behavior. The results of the mediation analysis are presented in Table 6.

In the first model, the findings indicated that attitude exerted a significant positive total effect (B = 0.363, *p* < 0.001) and a significant positive direct effect (B = 0.186, *p* = 0.012) on pro-environmental behavior. Furthermore, the indirect effect of attitude on pro-environmental behavior through implementation intention was statistically significant, as the 95% bootstrap confidence interval (LLCI = 0.100, ULCI = 0.273) did not include zero (Preacher & Hayes, 2004). These results indicate that implementation intention partially mediates the relationship between attitude and pro-environmental behavior. Therefore, Hypothesis 5 was supported.

In the second model, the findings revealed that behavioral intention had a significant positive total effect (B = 0.510, *p* < 0.001) and a significant positive direct effect (B = 0.330, *p* < 0.001) on pro-environmental behavior. In addition, the indirect effect of behavioral intention on pro-environmental behavior through implementation intention was found to be significant, as the 95% bootstrap confidence interval (LLCI = 0.072, ULCI = 0.299) excluded zero (Preacher & Hayes, 2004). These findings demonstrate that implementation intention partially mediates the relationship between pro-environmental behavioral intention and pro-environmental behavior. Accordingly, Hypothesis 6 was supported.

Given the cross-sectional design, these results represent statistical indirect associations and should not be interpreted as evidence of temporal or causal mediation.

#### 4.4.5. Moderating Effects of Environmental Knowledge and Situational Context

Hierarchical regression analysis was conducted to examine the moderating effects of environmental knowledge and situational context. For Hypothesis 7-1, the explanatory power of the model increased from 20.8% in Model 1 to 31.5% in Model 2 and 33.7% in Model 3. The interaction between attitude and environmental knowledge was statistically significant (B = 0.131, SE = 0.051, t = 2.563, *p* = 0.011, 95% CI [0.030, 0.232]), and the addition of the interaction term increased the explained variance by 2.2% (ΔR^2^ = 0.022, ΔF = 6.568, *p* = 0.011). Thus, environmental knowledge significantly moderated the relationship between attitude and implementation intention, supporting Hypothesis 7-1.

For Hypothesis 7-2, the interaction between behavioral intention and environmental knowledge was not statistically significant (B = 0.064, SE = 0.046, t = 1.390, *p* = 0.166, 95% CI [−0.027, 0.154]). Although R^2^ increased from 41.6% to 47.0% across the models, the additional variance explained by the interaction term was not significant (ΔR^2^ = 0.005, ΔF = 1.932, *p* = 0.166). Therefore, Hypothesis 7-2 was not supported.

For Hypothesis 8, the interaction between implementation intention and situational context was also not statistically significant (B = 0.050, SE = 0.060, t = 0.827, *p* = 0.409, 95% CI [−0.069, 0.168]). The interaction term explained only an additional 0.3% of the variance (ΔR^2^ = 0.003, ΔF = 0.684, *p* = 0.409). Thus, situational context did not significantly moderate the relationship between implementation intention and pro-environmental behavior, and Hypothesis 8 was not supported. Detailed results are presented in Table 7.

## 5. Discussion

This study examined the relationships among pro-environmental behavior–related variables using data from 204 Korean guests who had stayed at hotels and resorts in Gangwon State, one of South Korea’s major leisure destinations. Specifically, it investigated the relationships among attitude toward pro-environmental behavior, behavioral intention, implementation intention, and past pro-environmental behavior, as well as the moderating roles of environmental knowledge and situational context. The main findings are as follows.

First, hotel guests’ attitudes toward pro-environmental behavior were positively associated with both behavioral intention and implementation intention, while behavioral intention was also positively associated with implementation intention. Notably, behavioral intention showed a stronger association with implementation intention than attitude, suggesting that although favorable attitudes provide an important motivational foundation, a more proximal willingness to act may be more closely associated with the formation of specific situation-based plans. Furthermore, implementation intention was positively associated with pro-environmental behavior and accounted for significant indirect associations of both attitude and behavioral intention with behavior, highlighting its mediating role in the motivation-to-behavior process. Pro-environmental behavior was measured in this study as respondents’ self-reported behavior during their most recent hotel stay. In other words, favorable attitudes and a general willingness to act may be more strongly associated with pro-environmental behavior when accompanied by concrete, situation-specific implementation intentions. At the same time, the direct associations of attitude and behavioral intention with behavior remained statistically significant, indicating partial rather than complete statistical mediation. Thus, implementation intention should not be regarded as the sole mechanism connecting pro-environmental motivation with behavior, but rather as an important post-intentional mechanism through which general evaluations and behavioral willingness are indirectly associated with reported pro-environmental behavior. These findings are broadly consistent with previous studies highlighting the role of implementation intention as a post-intentional mechanism in research on the intention–behavior gap (Grimmer & Miles, 2017; Wang et al., 2021; Wang & Mangmeechai, 2021).

Second, environmental knowledge significantly moderated the relationship between attitude and implementation intention, whereas its moderating effect on the relationship between behavioral intention and implementation intention was not significant. Specifically, the positive association between attitude and implementation intention was stronger among respondents with higher levels of environmental knowledge. One possible explanation is that attitude reflects a general evaluation of pro-environmental behavior, but such a favorable evaluation may not by itself provide sufficient guidance for forming a concrete action plan. Environmental knowledge can serve as an action-relevant cognitive resource by helping individuals understand which environmental actions are appropriate and how they can be performed, thereby reducing uncertainty when translating favorable attitudes into implementation intentions. In contrast, behavioral intention represents a more proximal motivational readiness to act. Once individuals have already formed a relatively strong intention, they may possess sufficient motivational direction to develop implementation plans, leaving less additional moderating role for environmental knowledge. These findings partially support previous studies suggesting that environmental knowledge can strengthen relationships among variables related to pro-environmental behavior (Joshi & Rahman, 2015; Joseph, 2019; Chaihanchanchai & Anantachart, 2023). Given the cross-sectional design, these results should be interpreted as relationship-specific moderating patterns rather than as evidence of stage-specific effects in the behavior-formation process.

Third, situational context did not significantly moderate the relationship between implementation intention and pro-environmental behavior. This finding is consistent with Grimmer and Miles (2017) and indicates that, in the present sample, the strength of this association did not vary significantly according to the measured situational conditions. One possible explanation is that the hotel behaviors examined, such as turning off lights, reusing towels, and reducing water consumption, are relatively easy to perform and require limited external resources or support. Once a specific implementation plan has been formed, such behaviors may therefore be less dependent on variations in situational conditions. Another possibility is that the relatively homogeneous hotel context of the sample provided insufficient variation in situational conditions to detect a moderating effect. Importantly, the non-significant interaction should not be interpreted as evidence that situational context is unimportant or that implementation intention is necessarily more influential than contextual factors. Rather, the role of situational context may depend on the type of behavior and the extent to which its performance requires external opportunities, facilities, or resources.

## 6. Conclusions and Implications

### 6.1. Theoretical Implications

The theoretical implications of this study are as follows.

First, this study contributes to the literature by examining implementation intention as an important post-intentional construct in research on the intention–behavior gap. Previous studies based on the Theory of Planned Behavior have largely focused on the relationships of attitude and behavioral intention with behavior, while paying comparatively less attention to the planning processes involved in behavioral implementation (Carrington et al., 2010; Hassan et al., 2016). Drawing on Gollwitzer’s (1999) implementation intention theory and Carrington et al.’s (2010) intention–behavior gap model, this study examined the indirect associations of attitude and behavioral intention with pro-environmental behavior through implementation intention. In the present study, pro-environmental behavior was operationalized as respondents’ self-reported behavior during their most recent hotel stay. The findings showed that implementation intention was positively associated with pro-environmental behavior and accounted for significant indirect associations between both attitude and behavioral intention and this behavior measure. Thus, the study extends pro-environmental behavior research beyond an intention-centered approach by highlighting the relevance of specific post-intentional action planning.

Second, this study contributes to the literature by conceptualizing environmental knowledge not only as an antecedent of pro-environmental behavior but also as a moderating variable. Previous studies have mainly examined its direct relationships with pro-environmental attitudes, behavioral intentions, and pro-environmental behavior (M. Kim et al., 2018; Cuong, 2025), while relatively less attention has been paid to whether its moderating role differs across specific relationships. The findings showed that environmental knowledge strengthened the positive association between attitude and implementation intention, whereas it did not significantly moderate the association between behavioral intention and implementation intention. These results extend previous research by demonstrating that environmental knowledge may exhibit different moderating patterns across the relationships included in the proposed model.

Third, this study contributes to the literature by suggesting that pro-environmental behavior can be examined as an interconnected process involving motivational and post-intentional constructs rather than as a single behavioral outcome. The results showed significant associations among attitude, behavioral intention, implementation intention, and pro-environmental behavior. In addition, environmental knowledge exhibited different moderating patterns across specific relationships, strengthening the association between attitude and implementation intention but not the association between behavioral intention and implementation intention. These findings highlight the value of considering both motivational factors and post-intentional action planning when examining pro-environmental behavior. Therefore, by combining selected motivational constructs from the Theory of Planned Behavior with implementation intention theory, this study provides a post-intentional framework for examining hotel guests’ pro-environmental behavior.

### 6.2. Practical Implications

The practical implications of this study are as follows.

First, the significant positive associations of hotel guests’ attitudes toward pro-environmental behavior with both behavioral intention and implementation intention suggest that fostering favorable perceptions of pro-environmental practices may support guests’ willingness and planning to engage in such behavior. Therefore, hotels should communicate their environmental initiatives in visible and easily understandable ways. For example, information about energy-saving efforts, reductions in disposable amenities, towel and linen reuse programs, and local environmental protection activities can be communicated through hotel websites, social media, in-room materials, and the check-in process. Such communication can help guests recognize the environmental value of these practices and form more favorable evaluations of pro-environmental behavior.

Second, the significant positive associations of behavioral intention with implementation intention and of implementation intention with pro-environmental behavior suggest that hotels should help guests translate general pro-environmental intentions into specific action plans. Rather than relying on abstract messages such as “Please engage in pro-environmental behavior,” hotels can provide behavior-specific if–then cues at the point of action. For example, reminders near room exits can prompt guests to switch off unnecessary lights and heating or cooling systems when leaving the room; towel-reuse instructions can clearly indicate what guests should do if they wish to reuse their towels; and recycling prompts can be placed directly beside designated recycling bins. By linking specific situational cues with corresponding actions, such interventions can facilitate the formation and enactment of concrete implementation plans.

Third, the significant positive association between implementation intention and pro-environmental behavior suggests that hotels should create service conditions that make planned pro-environmental actions easy to carry out. To support the enactment of such plans, hotels can provide facilities and systems such as in-room power shut-off systems, clear recycling instructions, easily accessible recycling bins, towel and linen reuse options, and refillable amenities. These features can reduce practical barriers and make it easier for guests to perform the specific actions they intend to undertake. Therefore, pro-environmental practices should be designed to support the execution of guests’ implementation plans without creating unnecessary inconvenience.

Fourth, because environmental knowledge strengthened the positive association between attitude and implementation intention, hotels should provide action-relevant environmental information that helps guests translate favorable environmental attitudes into specific implementation plans. Rather than providing only general information about environmental issues, hotels can communicate both the environmental significance of a behavior and the specific action guests can take. For example, towel-reuse messages can briefly explain how reuse contributes to water and energy conservation while clearly indicating what guests should do if they wish to participate. Similar information can be provided for recycling and the reduction of disposable amenities. Thus, the managerial objective should not simply be to increase general environmental awareness, but to provide concise and actionable knowledge that supports the formation of specific pro-environmental plans.

Finally, because the moderating effect of situational context was not statistically significant, the present findings do not provide evidence that the measured situational conditions strengthened or weakened the association between implementation intention and pro-environmental behavior. This result does not imply that situational context is unimportant. Rather, hotels may benefit from focusing on behavior-specific enabling conditions that make planned pro-environmental actions easier to perform. For example, managers can provide accessible recycling bins, clearly communicated towel- and linen-reuse options, convenient refillable amenities, and visible energy-saving controls. Together with action-relevant environmental information and specific implementation cues, these facilities can support an environment in which guests can more easily carry out their intended pro-environmental actions.

### 6.3. Limitations and Suggestions for Future Research

This study has several limitations. First, because this study used self-reported survey data, the possibility of social desirability bias cannot be ruled out. Second, pro-environmental behavior was measured mainly through relatively easy-to-perform actions, such as turning off lights, reusing towels, and reducing the use of disposable amenities. Therefore, caution is needed when generalizing the findings to all types of pro-environmental behavior. Third, because the data were collected from hotel guests in domestic leisure destinations, the relative homogeneity of the sample may have limited the extent to which the effects of situational context were fully reflected. Fourth, the study employed a cross-sectional design. Behavioral intention and implementation intention were measured prospectively, whereas pro-environmental behavior was reported retrospectively. Consequently, the temporal ordering assumed in the conceptual model could not be established, and the indirect effects should not be interpreted as evidence of causal mediation.

Future research should combine self-reported measures with behavioral observation, experimental designs, or hotel-recorded behavioral data to improve measurement objectivity. It should also examine a wider range of pro-environmental behaviors, including high-involvement actions. To assess situational context more precisely, future studies should include guests from diverse accommodation settings that vary in hotel grade, accommodation type, and the strength of environmental policies and facilities. Finally, longitudinal or time-lagged designs are needed in which behavioral intention and implementation intention are measured before a hotel stay and behavior is subsequently observed or reported after the stay. Such designs would provide a stronger basis for examining the temporal relationships among intention, implementation planning, and pro-environmental behavior.

## Figures and Tables

**Table 1:** Measurement item descriptions.

Constructs	Code	Measurement Items (Shortened)
Attitude	AT1	Practicing pro-environmental behavior in hotels is good.
	AT2	Practicing pro-environmental behavior in hotels is worthwhile.
	AT3	Practicing pro-environmental behavior helps protect the environment.
	AT4	Practicing pro-environmental behavior is appropriate for hotel guests.
	AT5	Practicing pro-environmental behavior is worthwhile despite inconvenience.
Behavioral Intention	BI1	I intend to practice pro-environmental behavior in future hotel stays.
	BI2	I intend to make environmentally responsible choices in hotels.
	BI3	I am willing to cooperate with hotel environmental policies.
	BI4	I am willing to participate in hotel pro-environmental activities.
	BI5	I prefer hotels that actively implement environmental policies.
Implementation Intention	II1	If I leave my room, I will switch off unnecessary lights and HVAC.
	II2	If towels or linens are still usable, I will request that they not be replaced.
	II3	If disposable amenities are provided, I will use only what is necessary.
	II4	If I dispose of waste, I will follow the hotel’s recycling instructions.
	II5	If I use water during my hotel stay, I will avoid unnecessary consumption.
Pro-environmental Behavior	BH1	During my most recent stay, I switched off unnecessary lights and HVAC.
	BH2	During my most recent stay, I declined unnecessary towel or linen replacement.
	BH3	During my most recent stay, I avoided unnecessary disposable amenities.
	BH4	During my most recent stay, I sorted recyclable waste when possible.
	BH5	During my most recent stay, I avoided unnecessary water use.
Environmental Knowledge	EK1	I understand the environmental impacts of hotel operations.
	EK2	I know what environmentally friendly actions to take in hotels.
	EK3	I understand the environmental impact of disposable amenities.
	EK4	I understand that saving energy and water reduces environmental impacts.
	EK5	I can understand and follow hotel reuse and recycling instructions.
Situational Context	SC1	My companions cooperated in practicing pro-environmental behavior.
	SC2	My companions considered pro-environmental behavior important.
	SC3	I observed other guests practicing pro-environmental behavior.
	SC4	Hotel staff encouraged pro-environmental behavior.
	SC5	The hotel atmosphere encouraged pro-environmental behavior.

**Table 2:** Demographic characteristics of respondents (*n*= 204).

Category		*n*	%
Gender	Male	115	56.4
	Female	89	43.6
Age	20s	8	3.9
	30s	19	9.3
	40s	80	39.2
	50s	66	32.4
	60s and above	31	15.2
Education	High school diploma	14	6.9
	Bachelor’s degree	156	76.5
	Graduate degree	34	16.7
Companion	Alone	17	8.3
	Family	47	23.0
	Friends or partner	111	54.4
	Colleagues or group members	21	10.3
	Other	8	3.9
Monthly household income	<3 million KRW	19	9.3
	3 million–less than 5 million KRW	47	23.0
	5 million–less than 10 million KRW	106	52.0
	≥10 million KRW	32	15.7
Number of hotel stays per year	1–2 times	55	27
	3–5 times	101	49.5
	6–9 times	40	19.6
	≥10 times	8	3.9
Purpose of recent hotel stay	Tourism and leisure	125	61.3
	Business	30	14.7
	Events and conventions	17	8.3
	Family gathering	30	14.7
	Other	2	1.0
Total		204	100.0

**Table 3:** Results of Exploratory Factor Analysis (EFA).

Constructs	Code	Factor Loading	Eigenvalue	Variance Explained	Cronbach’s α
Attitude	AT1	0.849	9.990	37.001	0.936
	AT2	0.839			
	AT3	0.857			
	AT4	0.861			
	AT5	0.855			
Behavioral Intention	BI1	0.830	1.620	6.000	0.901
	BI3	0.742			
	BI4	0.743			
	BI5	0.742			
Implementation Intention	II1	0.768	2.475	9.165	0.902
	II2	0.796			
	II3	0.779			
	II4	0.713			
	II5	0.695			
Pro-environmental Behavior	BH1	0.820	3.431	12.708	0.926
	BH2	0.826			
	BH3	0.863			
	BH4	0.838			
	BH5	0.826			
Environmental Knowledge	EK2	0.795	1.212	4.487	0.865
	EK3	0.772			
	EK4	0.802			
	EK5	0.799			
Situational Context	SC1	0.814	1.945	7.205	0.897
	SC2	0.848			
	SC3	0.853			
	SC4	0.845			

Note. KMO = 0.919, Bartlett’s Test of Sphericity Chi-Square = 4011.957, df = 351, Sig. < 0.001.

**Table 4:** Correlation and discriminant validity analysis results.

	AVE	1	2	3	4	5	6
Attitude	0.726	**0.852**					
Behavioral Intention	0.586	0.471 **	**0.765**				
Implementation Intention	0.564	0.456 **	0.645 **	**0.751**			
Pro-environmental Behavior	0.697	0.345 **	0.465 **	0.449 **	**0.835**		
Environmental Knowledge	0.627	0.251 **	0.347 **	0.431 **	0.308 **	**0.792**	
Situational Context	0.706	0.095	0.300 **	0.326 **	0.256 **	0.448 **	**0.840**

Note. AVE = average variance extracted. Bold diagonal values represent the square roots of the AVE. ** *p* < 0.01, two-tailed.

**Table 5:** Results of regression analysis.

**A. Attitude and Behavioral Intention**								
**Dependent Variable**	**Independent Variable**	**B**	**SE**	**β**	**t**	***p***	**Tolerance**	**VIF**
	(Constant)	2.437	0.298		8.190	<0.001		
Behavioral Intention	Attitude	0.452	0.060	0.471	7.593	<0.001	1.000	1.000
R^2^ = 0.222, Adj R^2^ = 0.218, F = 57.658, Sig. < 0.001								
**B. Attitude, Behavioral Intention, and Implementation Intention**								
**Dependent Variable**	**Independent Variable**	**B**	**SE**	**β**	**t**	***p***	**Tolerance**	**VIF**
	(Constant)	1.423	0.276		5.157	<0.001		
Implementation Intention	Attitude	0.178	0.054	0.196	3.289	0.001	0.778	1.285
	Behavioral Intention	0.525	0.057	0.553	9.285	<0.001	0.778	1.285
R^2^ = 0.446, Adj R^2^ = 0.440, F = 80.861, Sig. < 0.001								
**C. Implementation Intention and Behavior**								
**Dependent Variable**	**Independent Variable**	**B**	**SE**	**β**	**t**	***p***	**Tolerance**	**VIF**
	(Constant)	1.923	0.351		5.478	<0.001		
Pro-environmental Behavior	Implementation Intention	0.518	0.073	0.449	7.140	<0.001	1.000	1.000
R^2^ = 0.201, Adj R^2^ = 0.198, F = 50.973, Sig. < 0.001								

**Table 6:** Results of mediating effect analysis.

	B	SE	t	*p*	LLCI	ULCI
**Total Effect**						
Attitude → Pro-environmental Behavior	0.363	0.069	5.226	<0.001	0.226	0.500
Intention → Pro-environmental Behavior	0.510	0.068	7.474	<0.001	0.375	0.645
**Direct Effect**						
Attitude → Pro-environmental Behavior	0.186	0.073	2.542	0.012	0.042	0.331
Intention → Pro-environmental Behavior	0.330	0.087	3.780	<0.001	0.158	0.502
**Indirect Effect**						
Attitude → Pro-environmental Behavior	0.177	0.044	-	-	0.100	0.273
Intention → Pro-environmental Behavior	0.180	0.058	-	-	0.072	0.299

**Table 7:** Results of moderating effect analysis. (**A**) Moderating effect of environmental knowledge on the relationship between attitude and implementation intention (H7-1). (**B**) Moderating effect of environmental knowledge on the relationship between behavioral intention and implementation intention (H7-2). (**C**) Moderating effect of situational context on the relationship between implementation intention and pro-environmental behavior (H8).

**(A) Moderating effect of environmental knowledge on the relationship between attitude and implementation intention (H7-1).**											
**Model**	**Predictor**	**B**	**SE**	**t**	***p***	**95% CI**	**VIF**	**R^2^**	**ΔR^2^**	**ΔF**	**p(ΔF)**
1	Attitude	0.416	0.057	7.287	<0.001	[0.303, 0.528]	1.000	0.208	0.208	53.103	<0.001
2	Attitude	0.339	0.055	6.162	<0.001	[0.230, 0.447]	1.067	0.315	0.107	31.277	<0.001
	Environmental Knowledge	0.382	0.068	5.593	<0.001	[0.247, 0.516]	1.067				
3	Attitude	0.342	0.054	6.305	<0.001	[0.235, 0.449]	1.068	0.337	0.022	6.568	0.011
	Environmental Knowledge	0.412	0.068	6.024	<0.001	[0.277, 0.546]	1.099				
	Attitude × Environmental Knowledge	0.131	0.051	2.563	0.011	[0.030, 0.232]	1.035				
**(B) Moderating effect of environmental knowledge on the relationship between behavioral intention and implementation intention (H7-2).**											
**Model**	**Predictor**	**B**	**SE**	**t**	***p***	**95% CI**	**VIF**	**R^2^**	**ΔR^2^**	**ΔF**	**p(ΔF)**
1	Behavioral Intention	0.612	0.051	11.996	<0.001	[0.512, 0.713]	1.000	0.416	0.416	143.912	<0.001
2	Behavioral Intention	0.535	0.052	10.237	<0.001	[0.432, 0.638]	1.137	0.465	0.049	18.210	<0.001
	Environmental Knowledge	0.266	0.062	4.267	<0.001	[0.143, 0.389]	1.137				
3	Behavioral Intention	0.530	0.052	10.139	<0.001	[0.427, 0.633]	1.143	0.470	0.005	1.932	0.166
	Environmental Knowledge	0.288	0.064	4.490	<0.001	[0.162, 0.415]	1.215				
	Behavioral Intention × Environmental Knowledge	0.064	0.046	1.390	0.166	[−0.027, 0.154]	1.069				
**(C) Moderating effect of situational context on the relationship between implementation intention and pro-environmental behavior (H8).**											
**Model**	**Predictor**	**B**	**SE**	**t**	***p***	**95% CI**	**VIF**	**R^2^**	**ΔR^2^**	**ΔF**	**p(ΔF)**
1	Implementation Intention	0.518	0.073	7.140	<0.001	[0.375, 0.661]	1.000	0.201	0.201	50.973	<0.001
2	Implementation Intention	0.472	0.076	6.183	<0.001	[0.321, 0.622]	1.119	0.215	0.014	3.480	0.064
	Situational Context	0.144	0.077	1.865	0.064	[−0.008, 0.297]	1.119				
3	Implementation Intention	0.463	0.077	6.007	<0.001	[0.311, 0.615]	1.140	0.218	0.003	0.684	0.409
	Situational Context	0.164	0.081	2.023	0.044	[0.004, 0.323]	1.220				
	Implementation Intention × Situational Context	0.050	0.060	0.827	0.409	[−0.069, 0.168]	1.092				

Note. Predictors and moderators were mean-centered. B = unstandardized coefficient; SE = standard error; CI = confidence interval; VIF = variance inflation factor.

## Data Availability

The data presented in this study are available from the corresponding author upon reasonable request.
